# Evaluation of Interactive Effect of Anti-Skid Performance of Iron Tailings Sand Asphalt Mixture Under Coupling Effect

**DOI:** 10.3390/ma19071378

**Published:** 2026-03-30

**Authors:** Zhiqiao Cheng, Liwenze He, Xiaoyan Liu, Xiu Luo, Yixin Lu, Jiao Chen

**Affiliations:** 1 Department of Civil Engineering, Chengdu Technological University, Yibin 644000, China; 2 School of Materials and Environmental Engineering, Chengdu Technological University, Chengdu 611730, China

**Keywords:** iron tailings sand, asphalt mixture, anti-skid performance, correlation model, accelerated wear and tear

## Abstract

To achieve the resource utilization of iron tailings sand and improve the skid resistance of asphalt pavement, this study takes asphalt mixtures with different contents of iron tailings sand replacing partial fine aggregates as research objects. Through accelerated wear tests, the skid resistance performance was systematically evaluated under the coupled effects of iron tailings sand content, ambient temperature and wear cycles. The variation laws of the British Pendulum Number (BPN) and Mean Texture Depth (MTD) of the mixtures were investigated, and the mechanism and influence characteristics of various factors on skid resistance were further interpreted in combination with correlation heatmap analysis. The results show that the mixture with 60% iron tailings sand content maintains relatively high initial and final attenuation values of both BPN and MTD, which can effectively delay the degradation of skid resistance under long-term wear, thus representing the preferred content for engineering applications. Temperature is the core environmental factor affecting skid resistance: high temperature accelerates performance degradation, while the mixtures exhibit more stable skid resistance under medium- and low-temperature conditions. The coupling of high iron tailings content and high temperature produces adverse interaction effects, leading to performance differentiation. The relevant quantitative analysis and fitting models enable the long-term prediction of skid resistance, providing support for pavement maintenance decision making.

## 1. Introduction

With the continuous improvement in China’s highway network and the steady growth of traffic volume, the skid resistance of pavements has become an increasingly critical guarantee for driving safety, which is directly related to the safety and reliability of road operation [1]. Under complex service conditions such as icy and snowy weather [2], rainwater accumulation [3], and the presence of attachments [4] and contaminants [5] on the pavement, the attenuation of pavement skid resistance is prone to cause potential safety hazards such as vehicle brake failure and sideslip, seriously endangering the lives and property of drivers and passengers. As a large amount of solid waste generated during mining, iron tailings sand has an enormous cumulative stockpile. It features high hardness [6,7], a rough surface and excellent angularity [8,9]. Replacing part of traditional aggregates with iron tailings sand in the preparation of asphalt mixtures [10,11] can not only specifically improve pavement skid resistance [12] and solve the problem of insufficient skid resistance durability of traditional mixtures, but also promote the resource utilization of iron tailings sand, reduce the environmental pressure caused by solid waste stockpiling, and deeply align with the era requirements of green, low-carbon development and resource recycling in the transportation field, thus possessing important engineering application value and environmental benefits [13].

Scholars at home and abroad have carried out extensive systematic research on the skid resistance of asphalt pavements, forming a multi-dimensional research system covering skid resistance mechanisms, influencing factors, evaluation methods and optimization measures [14]. In terms of skid resistance mechanisms, existing studies generally hold that the skid resistance of pavements mainly depends on the macro- and micro-texture characteristics of the pavement surface [15]: micro-texture determines the frictional performance under low-adhesion conditions, while macro-texture dominates drainage capacity and long-term skid resistance stability. The two act synergistically to ensure the pavement skid resistance effect, and the texture morphology has self-similar fractal characteristics, with a significant correlation between its fractal dimension and skid resistance indicators [16]. In the research on influencing factors, scholars have identified material properties, traffic loads, environmental conditions and construction processes as the core influencing factors [17,18]. Among them, aggregate hardness, angularity and wear resistance directly determine texture stability [19]; long-term wear from traffic loads leads to the gradual smoothing of textures and attenuation of skid resistance; environmental factors such as high temperature, rainfall and freeze–thaw accelerate asphalt aging and aggregate spalling [20,21], further exacerbating the degradation of skid resistance, and there is an obvious coupling effect among various factors [22]. In terms of evaluation and detection, an evaluation index system centered on friction coefficient and texture depth has been formed [23], and a combination of traditional and modern detection methods such as pendulum friction testing and laser vision measurement have been developed. Among them, the non-contact detection technology that collects texture data with laser sensors combined with intelligent analysis models has greatly improved the efficiency and accuracy of skid resistance assessment, and the prediction models constructed in some studies can achieve the accurate prediction of the attenuation trend of skid resistance [24,25]. In terms of optimization measures, scholars have proposed a variety of technical approaches, including the selection of high-angularity and high-hardness anti-skid aggregates, optimization of asphalt mixture gradation design, adoption of anti-skid surface layer structures, and improvement in the adhesion between asphalt and aggregates by adding admixtures, which provide abundant theoretical and technical support for improving the skid resistance of asphalt pavements [26,27].

On the whole, early studies mostly focused on the anti-polishing characteristics of natural aggregates such as basalt, limestone and granite [28]. In recent years, under the trend of solid waste resource utilization in road engineering, iron tailings sand, as a large amount of solid waste from mining, has tried to replace part of aggregates in the preparation of asphalt mixtures due to its high hardness and rich surface texture [29]. However, most studies only focus on the influence of different contents of iron tailings sand on the road performance of mixtures, lacking the systematic analysis of the macro- and micro-texture, frictional performance and skid resistance durability of mixtures after replacing fine aggregates with different contents of iron tailings sand. Moreover, the internal correlation between different contents of iron tailings sand and the skid resistance of mixtures as well as the optimal mixing range have not been clarified yet.

In view of this, this paper takes asphalt mixtures with iron tailings sand replacing part of fine aggregates as the research object. Through accelerated loading wear tests, it systematically investigates the variation rules of BPN and MTD values of iron tailings sand asphalt mixtures under different wear cycles, with the coupling effects of various iron tailings sand dosages (0%, 20%, 40%, 60%, 80%, 100%) and different environmental temperatures (20 °C, 40 °C, 60 °C). The influence mechanism of iron tailings sand dosage, temperature and wear on skid resistance is revealed, and the critical deterioration nodes of skid resistance are identified. Furthermore, this study attempts to propose an optimal blending range that balances skid resistance and resource utilization, so as to provide theoretical support and technical reference for the engineering application of iron tailings sand in the anti-skid surface layer of asphalt pavements.

## 2. Materials and Methods

### 2.1. Materials

In this study, basalt was used as the coarse aggregate, with its technical properties listed in Table 1. Limestone was adopted as the fine aggregate, with its technical properties presented in Table 2. 70# (Class A) base asphalt was selected, and the modified asphalt was SBS I-D modified asphalt with a dosage of 4.5%. The technical properties of the asphalt binders are summarized in Table 3.

The iron tailings sand used in this study presents an overall grayish black color, and iron tailings particles with a particle size between 0.075 mm and 4.75 mm are selected through screening and sorting. The main technical indicators are shown in Table 4.

The experiment used SMA-13 asphalt mixture, and in this study, iron tailings sand was used instead of 0.075–4.75 mm limestone fine aggregate. The proportion of iron tailings sand was 0, 20%, 40%, 60%, 80%, and 100% of the volume of fine aggregate, respectively. Due to the different densities and significant differences between iron tailings sand and limestone, volume control is adopted in the grading design of the mixture. Based on the principle of equal volume replacement, the volume method is used to design the grading composition of asphalt mixtures with different volume contents of iron tailings sand. The pre-configured mineral materials are separated into fine aggregate parts with a 4.75 mm aperture, and then 20%, 40%, 60%, 80%, and 100% of the fine aggregate volume is replaced with iron tailings sand. The optimal oil stone ratios for SMA-13 asphalt mixture with six different dosages of iron tailings sand are 4.5%, 4.5%, 4.6%, 4.7%, 4.8%, and 4.9%, respectively.

### 2.2. Test Method

#### 2.2.1. Marshall Test

The proportion of iron tailings sand is 0 (reference group), 20%, 40%, 60%, 80%, and 100% of the volume of fine aggregate, respectively. The gradation of the mixture is shown in Table 5. According to the requirements of the “Test Code for Asphalt and Asphalt Mixtures in Highway Engineering” (JTG E20-2011) [30], Marshall tests were conducted to obtain the optimal oil stone ratio and Marshall volume index, as shown in Table 6.

#### 2.2.2. Skid Resistance Test

**Figure 1:**
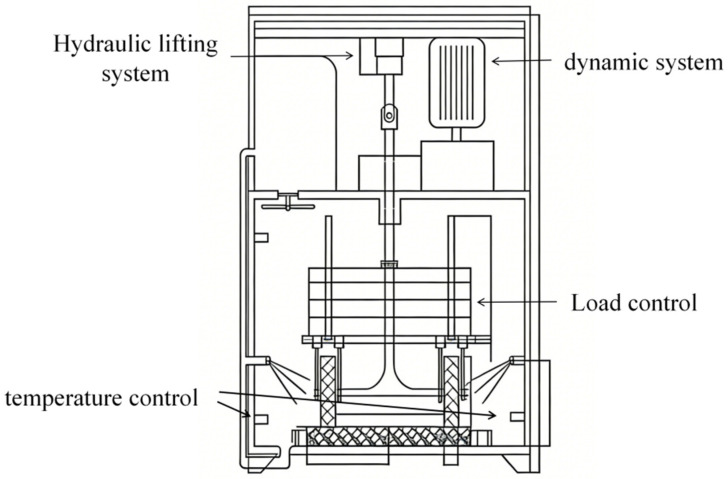
Accelerated wear equipment.

According to the test procedures specified in Field Test Regulations for Highway Subgrade and Pavement (JTG 3450-2019) [31], rutting plate specimens (300 × 300 × 50 mm) of SMA-13 asphalt mixture with different iron tailings sand contents were prepared. Accelerated wear tests were carried out using a self-developed small-scale accelerated abrasion device, as shown in Figure 1. The wheel load was 0.7 MPa. The abrasion tester was equipped with two rubber wheels with a diameter of 200 mm and a width of 50 mm, operating at a frequency of 3000 r/h. Water was sprayed onto the specimen surface during the accelerated abrasion process.

The test temperature was strictly controlled. All specimens were conditioned at the target test temperatures of 20 °C, 40 °C, and 60 °C for 4 h to ensure uniform and stable internal temperature. Meanwhile, the accelerated abrasion device was equipped with a thermostatic chamber, which was heated and insulated by built-in heating modules and circulating air systems to maintain the ambient temperature consistent with the set test temperature. Tests were initiated only after the device temperature was stabilized.

The British Pendulum Number (BPN) of the specimen surface was measured using a pendulum friction tester (ZBBM-III, Jiangsu Muyang Zhongke Highway Instrument Co., Ltd., Yangzhou, China). The lifting handle and base screws were adjusted to ensure that the sliding distance of the rubber slider between two consecutive contacts was exactly 126 mm, which is critical to guarantee the accurate conversion of potential energy. The rubber slider had a hardness of 56 (Shore A), meeting the specification requirements. Water was sprayed on the specimen surface during BPN testing.

The Mean Texture Depth (MTD) was determined by the sand patch method.

All skid resistance indicators were tested in triplicate to ensure stability and repeatability. The average value of three tests was adopted as the final result on the premise that the coefficient of variation (COV) of each group met the required criteria.

The BPN test was repeated on the same rutting board, and the wear cycle times were repeated. MTD used the sanding method for testing, and to avoid sand and gravel entering the interior of the specimen and being unable to be completely cleaned, it was not repeated on the same rutting board. MTD used the parallel rutting plates of the same batch, same mix ratio, and synchronous forming for testing to ensure the reliability of test results and data.

## 3. Results and Discussion

### 3.1. Anti-Slip Indicators Under Different Iron Tailings Sand Dosages

**Figure 2:**
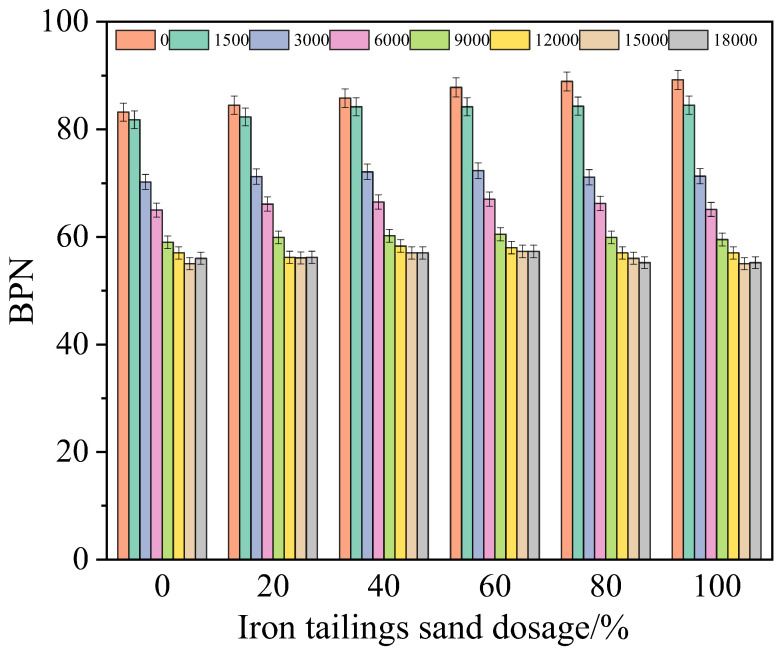
BPN evolution trend under different iron tailings sand content.

**Figure 3:**
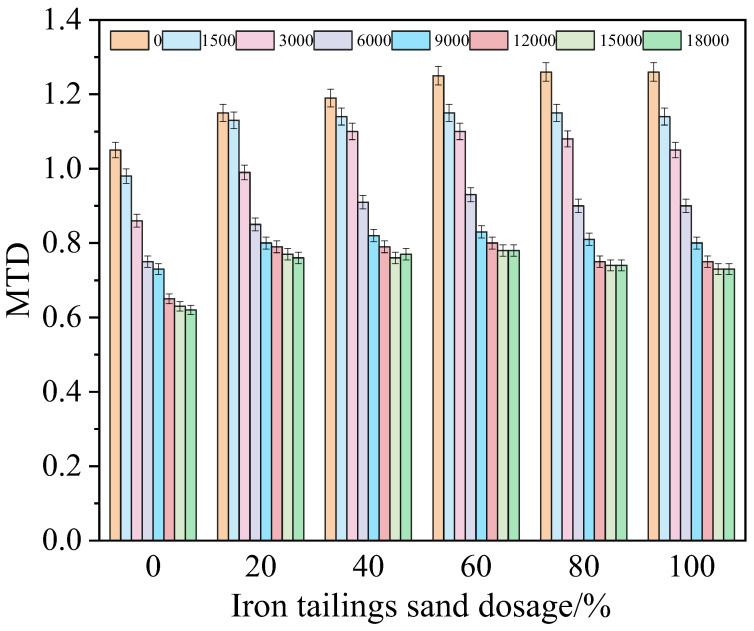
MTD evolution trend under different iron tailings sand content.

To investigate the influence of replacing part of fine aggregates with iron tailings sand on the skid resistance of SMA-13 asphalt mixtures, six types of SMA-13 asphalt mixture rut plate specimens with iron tailings sand contents of 0, 20%, 40%, 60%, 80% and 100% were prepared, respectively. An indoor accelerated wear device was used to conduct accelerated wear tests, and the British Pendulum Number (BPN) and Mean Texture Depth (MTD) values under different wear times were tested separately. The results are shown in Figure 2 and Figure 3.

As shown in Figure 2, the asphalt mixtures with different iron tailings sand contents exhibit differentiated performance rules. In the initial state, the BPN value of the specimen with 100% iron tailings sand content is 7.21% higher than that of the reference group (0% content), indicating that the angular particle morphology of iron tailings sand can significantly improve the initial skid resistance of the mixture. With the accelerated wear times increasing to 18,000 times, the BPN values of all specimens with different contents show attenuation, but the final BPN value of the specimen with 100% iron tailings sand content is only 1.43% lower than that of the reference group, which indicates that the high content of iron tailings sand has little impact on the long-term final value of the skid resistance of the mixture. It is worth noting that the BPN values of specimens with different contents after 18,000 times of wear are either higher or lower than that of the reference group, revealing that the content of iron tailings sand and skid resistance are not in a simple positive correlation—that is, the higher the content, the better the performance—and there exists an optimal content range. This result shows that iron tailings sand can not only effectively improve the initial skid resistance level, but also maintain the skid resistance equivalent to that of the reference group after long-term wear, providing an experimental basis for the reasonable mixing of iron tailings sand in the anti-skid layer of asphalt pavements.

It is worth noting that the specimen with 60% content exhibits excellent evolutionary characteristics of skid resistance. Not only its initial BPN value is significantly higher than that of the reference group, but also the final BPN value remains at a relatively high level among all content groups and is better than that of the reference group after 18,000 times of accelerated wear. This indicates that an appropriate content of iron tailings sand can effectively delay the deterioration of the skid resistance of the mixture under long-term wear, balancing the initial skid resistance level and long-term skid resistance stability.

As shown in Figure 3, with the increase in accelerated wear cycles, the Mean Texture Depth (MTD) values of asphalt mixtures with different iron tailings sand contents all present a gradual attenuation trend; however, the incorporation of iron tailings sand significantly changes the initial level of texture depth and the long-term attenuation law. In the initial state, the MTD value of the specimen with 100% iron tailings sand content is 20.0% higher than that of the reference group (0% content). This phenomenon is due to the angular coarse particle morphology of iron tailings sand, which can form richer macro-textural structures on the surface of the mixture. After 18,000 accelerated wear cycles, the final MTD value of the specimen with this content is still 17.74% higher than that of the reference group, indicating that high-content iron tailings sand can not only improve the initial texture depth, but also effectively slow down the texture deterioration rate under wear action. Further observation of the evolution process of each content group shows that the incorporation of iron tailings sand can significantly enhance the wear resistance of the surface texture of asphalt mixtures. Its hard crystal structure can better maintain the integrity of macro-textures under long-term cyclic loads, and this characteristic provides key structural performance support for the application of iron tailings sand in asphalt pavements with high wear resistance requirements.

It is worth noting that the asphalt mixture specimen with 60% iron tailings sand content exhibits more excellent evolutionary characteristics of skid resistance. The initial MTD value of the specimen under this content is significantly higher than that of the reference group, and after 18,000 accelerated wear cycles, the final MTD value still remains at a relatively high level, which is superior to that of the specimens with 80% and 100% contents. This phenomenon indicates that, although the specimens with high contents of 80% and 100% have better initial texture depth, the deterioration rate of their surface textures under long-term wear is faster. In contrast, 60% content can effectively delay the attenuation of macro-textures, achieving a balance between the initial skid resistance level and long-term wear resistance.

### 3.2. Anti-Skid Indexes Under Different Temperatures

**Figure 4:**
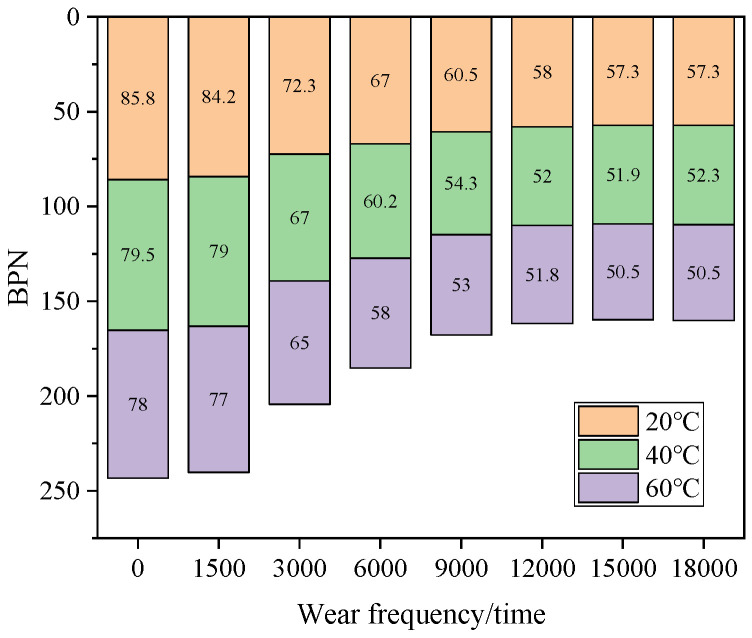
BPN values at different temperatures.

**Figure 5:**
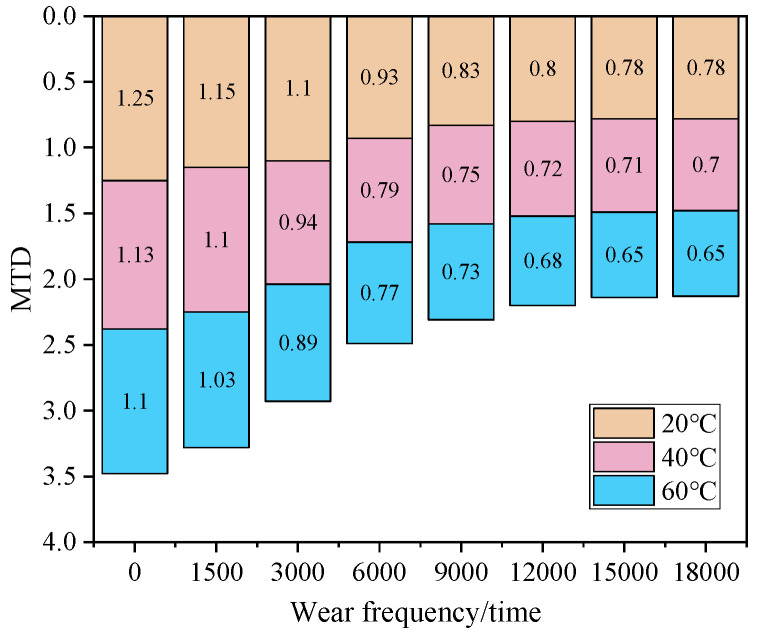
MTD values at different temperatures.

Based on the above analysis of the skid resistance evolution of asphalt mixtures with different iron tailings sand contents under accelerated wear, the 60% content has been initially verified to achieve the optimal balance between the initial skid resistance level and long-term wear resistance stability. To further explore the skid resistance performance of the mixture with this content in the actual service environment, the skid resistance index tests under different temperature gradients will be carried out for the asphalt mixture with 60% iron tailings sand content. In this study, three test conditions (20 °C, 40 °C and 60 °C) were selected to clarify the influence law of temperature effect on its skid resistance performance, and the results are shown in Figure 4 and Figure 5.

As shown in Figure 4, from the perspective of the BPN evolution characteristics of the asphalt mixture with 60% iron tailings sand content at different temperatures, with the increase in accelerated wear cycles, the BPN values under the three temperature conditions all show a continuous attenuation trend, but the influence of temperature on the attenuation rate and amplitude is relatively significant. In the initial state, the BPN value is the highest at 20 °C (85.8) and the lowest at 60 °C (78), indicating that high temperature directly weakens the initial skid resistance of the mixture. When the wear cycles increase to 18,000 times, the BPN value at 60 °C (59.3) decreases by about 24.0% compared with the initial value, while the BPN value at 20 °C (73.3) only decreases by about 14.6%. This indicates that the high-temperature environment accelerates the deterioration of the aggregate surface texture and significantly speeds up the attenuation rate of skid resistance. It is worth noting that, at each wear stage, the BPN values under 20 °C and 40 °C are always at a high level with relatively gentle attenuation amplitude. This result reveals that the mixture with 60% iron tailings sand content has more stable long-term skid resistance in medium- and low-temperature environments, while in high-temperature environments, surface modification and other methods are needed to further improve its skid resistance durability, providing targeted technical basis for the engineering application of this content scheme in different climate zones.

As shown in Figure 5, with the increase in accelerated wear cycles, the MTD values under the three temperature conditions all show a continuous attenuation trend, and the influence of temperature on the attenuation rate presents an obvious regularity. In the initial state, the MTD value is the highest at 20 °C (1.25) and the lowest at 60 °C (1.1). This phenomenon can be initially attributed to the decrease in the adhesion of asphalt binder at high temperatures, which weakens the initial supporting effect of the aggregate surface texture. When the wear cycles increase to 18,000 times, the MTD value at 60 °C (0.65) decreases by about 78.3% compared with the initial value, while the MTD value at 20 °C (0.78) only decreases by about 77.1%. The more significant MTD attenuation under high-temperature environment is speculated to be related to two factors: first, the overall stiffness of asphalt mixture decreases at high temperatures, making it easier for micro-displacement and migration of aggregate particles under wear load, which destroys the stability of the original macro-texture; second, high temperature softens the asphalt film, reduces its wrapping force on aggregate particles, and accelerates the wear and spalling of surface texture.

It is worth noting that, at each wear stage, the MTD value at 20 °C is always at the highest level with relatively gentle attenuation amplitude, while the MTD value at 60 °C has the largest attenuation amplitude. This result reveals that the mixture with 60% iron tailings sand content can better maintain the integrity of macro-texture in medium- and low-temperature environments and has a more stable long-term texture depth. The above conjecture about aggregate migration and asphalt softening at high temperatures needs to be further verified through microscopic observation and mechanical simulation in subsequent studies, so as to provide more accurate technical support for the engineering application of this content scheme in different climate zones.

### 3.3. Skid Resistance Decay Law of Iron Tailings Sand Asphalt Mixtures

**Figure 6:**
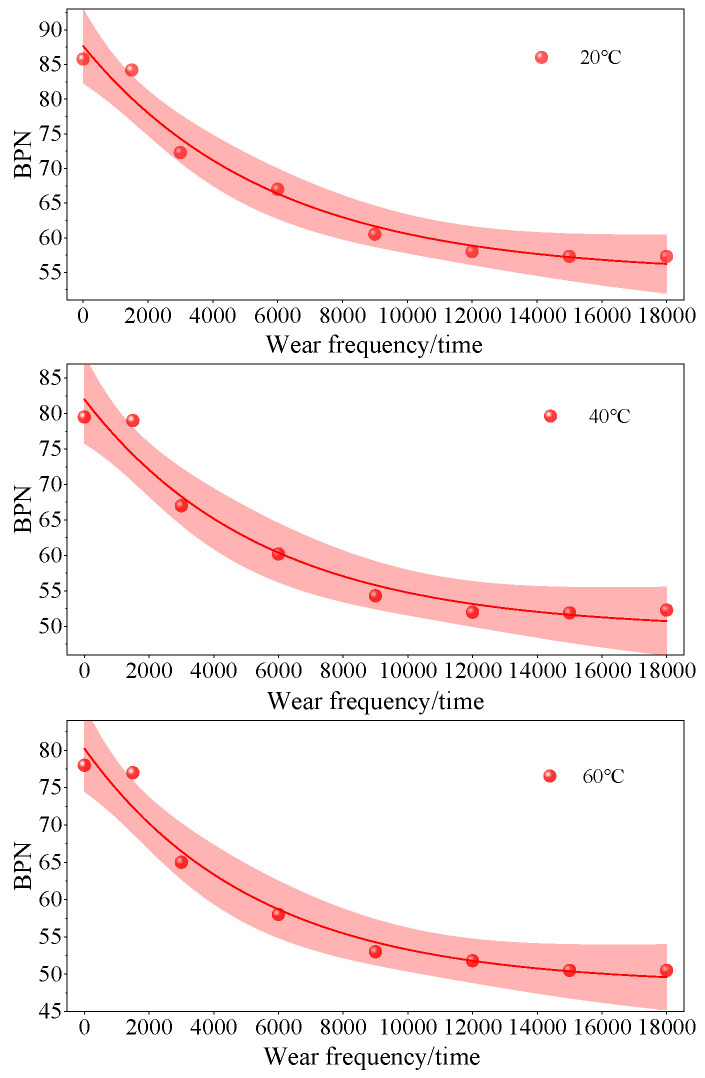
Decay model of BPN values under different temperatures and wear cycles.

**Figure 7:**
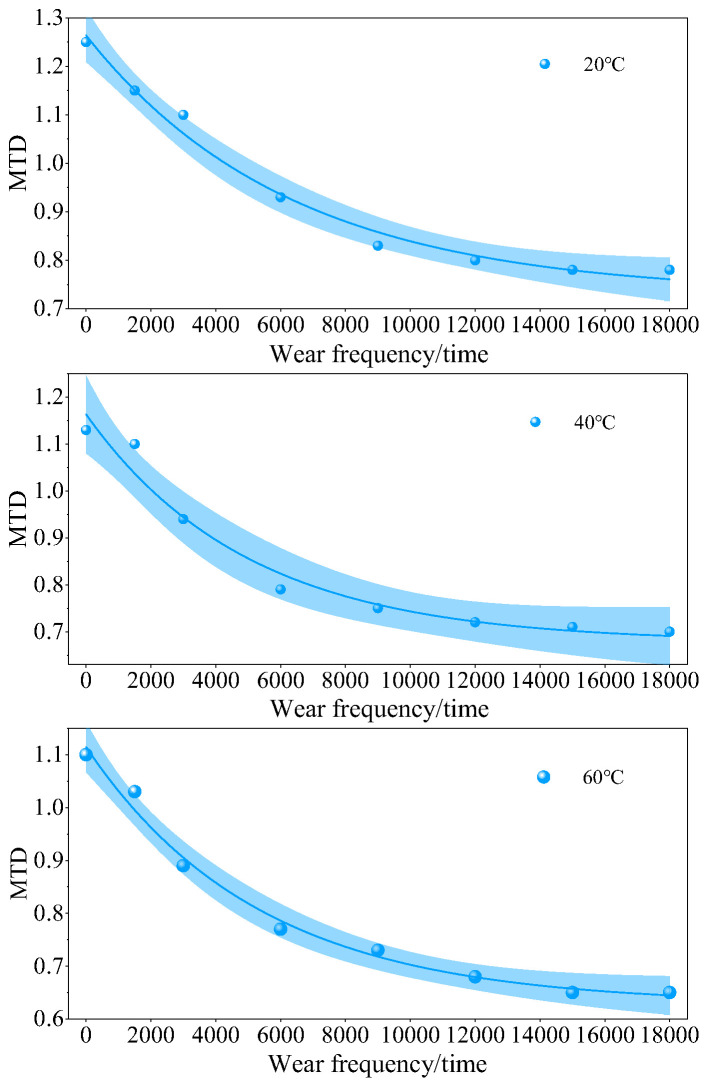
Decay model of MTD values under different temperatures and wear cycles.

Based on the above indoor accelerated wear test results, the YIdFert1 model was used to fit and analyze the changes in BPN values and MTD of SMA-13 iron tailings sand asphalt mixture with 60% dosage under different temperatures with wear cycles. The expression of the YIdFert1 model is y = a + be^−kx^, where *a* is the final attenuation value, *b* is the attenuation amplitude, and *k* is the attenuation rate. The fitting curves are shown in Figure 6 and Figure 7.

At the three temperatures, the attenuation patterns of BPN and MTD with the number of abrasions can be well fitted by a nonlinear model, with a narrower 95% confidence band overall, indicating high reliability of the model. Among them, the confidence bands for both indicators are the narrowest at 20 °C, with minimal data dispersion and the most stable attenuation patterns for skid resistance and texture performance. As the temperature increases, both initial BPN and MTD decrease, and the attenuation amplitude increases, with different indicators exhibiting varying sensitivities to temperature: the confidence band for BPN gradually widens with increasing temperature, indicating that high temperatures continue to exacerbate the variability of the skid resistance abrasion process; however, the confidence band for MTD only significantly expands at 40 °C, approaching the level at 20 °C at 60 °C, suggesting that 40 °C may be the viscoelastic transition zone for the texture abrasion process, leading to the highest uncertainty in fitting at this temperature. The confidence bands of all curves expand to varying degrees at both ends of the abrasion range, indicating that predictions under extreme abrasion conditions need to be cautious.

As shown in Figure 6, the YIdFert1 model was adopted to fit the decay law of BPN values of asphalt mixture with 60% iron tailings sand dosage under different temperatures. The fitting results show that the correlation coefficient R^2^ at 20 °C, 40 °C, and 60 °C is 0.97, 0.96, and 0.96, respectively, all higher than 0.95. Moreover, the results of F-test indicate that *p* is much less than 0.05, which demonstrates that the model can accurately describe the attenuation process of BPN values of the mixture with 60% iron tailings sand dosage and has good statistical significance.

Based on the temperature sensitivity characteristics of asphalt mixtures, we speculate that the softening of asphalt binder and the decrease in aggregate particle adhesion at high temperatures are the core mechanisms leading to accelerated degradation of anti-skid performance. However, this is only our hypothesis. This model not only achieves the quantitative prediction of BPN value decay at different temperatures, but also provides a quantitative tool for exploring the degradation mechanism of anti-skid performance under temperature wear coupling in the future. Its fitting parameters can be directly applied to the long-term prediction model of road anti-skid performance, providing theoretical support for the engineering application of 60% iron tailings sand mixture in different climate zones.

As shown in Figure 7, the same YIdFert1 model used for BPN decay analysis was employed to fit the MTD decay laws of the asphalt mixture with 60% iron tailings sand content at different temperatures. The results show that the correlation coefficient R^2^ at 20 °C, 40 °C and 60 °C is 0.98, 0.96 and 0.98, respectively, all above 0.95. In addition, the F-test results indicate that *p* = 0.001, which is much less than 0.05, demonstrating that the model can also accurately characterize the MTD attenuation process of the mixture and presents satisfactory statistical reliability.

Similar to the fitting characteristics of BPN values, the final attenuation value *a* of MTD values shows a decreasing trend with the increase in temperature, and the attenuation rate *k* increases with the increase in temperature. This reveals that the high-temperature environment not only reduces the stable residual level of texture depth, but also accelerates the deterioration rate of macro-textures. The difference is that the overall fitting correlation coefficient of MTD values is slightly higher than that of BPN values, indicating that the model has a higher explanatory power for the attenuation process of texture depth. This difference stems from the subtle differences in the evolution mechanisms between the macro-texture deterioration reflected by MTD and the micro-texture wear reflected by BPN: the attenuation of MTD is mainly dominated by the displacement and spalling of aggregate particles, and its evolution law is more consistent with the exponential attenuation characteristics.

Combined with the previous experimental phenomena, we infer that the softening of asphalt binder and the reduced adhesion between aggregate particles at high temperatures remain the dominant mechanisms responsible for the accelerated decay of MTD values, which is consistent with the degradation mechanism of BPN values. It should be emphasized that this is only our hypothesis. The parameters obtained from the model fitting can not only be used for the long-term prediction of Mean Texture Depth at different temperatures, but also complement the fitting results of BPN values. This provides multi-dimensional quantitative evidence for comprehensively revealing the skid resistance degradation mechanism of the mixture with 60% iron tailings sand content under the coupled effects of temperature and abrasion, thereby offering more systematic theoretical support for the engineering application of this dosage scheme in different climatic regions.

### 3.4. High-Temperature Performance Test

**Figure 8:**
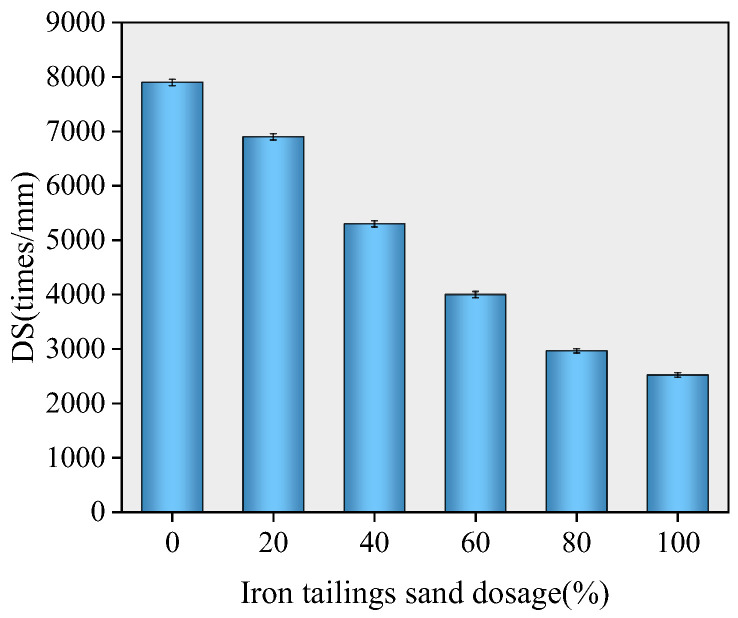
Dynamic stability at different dosages.

To further investigate the high-temperature performance of iron tailings sand asphalt mixtures, wheel tracking tests were conducted on specimens with different iron tailings sand contents. The dynamic stability (DS) under various replacement levels is presented in Figure 8.

The dynamic stability (DS) of SMA-13 mixtures with different iron tailings sand contents shows a continuous decreasing trend with the increase in replacement level. DS represents the resistance of asphalt mixtures to permanent deformation at high temperatures, and a higher value indicates superior high-temperature performance. At 0% replacement, the DS reaches its maximum value, indicating the optimal high-temperature performance. As the replacement level gradually increases to 20%, 40%, and 60%, the DS decreases accordingly but still meets the specification requirements. When the replacement level rises to 80%, the DS decreases to 2965 cycles/mm, which no longer satisfies the requirement that the dynamic stability of asphalt mixtures for expressways should be no less than 3000 cycles/mm. At 100% replacement, the DS further drops to 2523 cycles/mm, and the deterioration of high-temperature performance becomes more significant. It indicates that a high content of iron tailings sand can significantly weaken the high-temperature stability of the mixture.

### 3.5. Correlation Model Between Iron Tailings Sand Content-Abrasion Frequency and Anti-Sliding Index

#### 3.5.1. Correlation Model of Iron Tailings Sand Content-Abrasion Frequency and BPN

**Figure 9:**
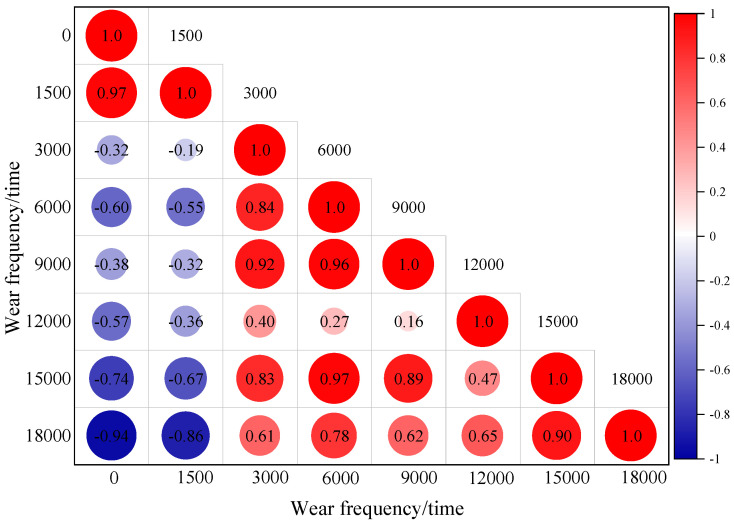
Correlation model of BPN values under different wear cycles.

**Figure 10:**
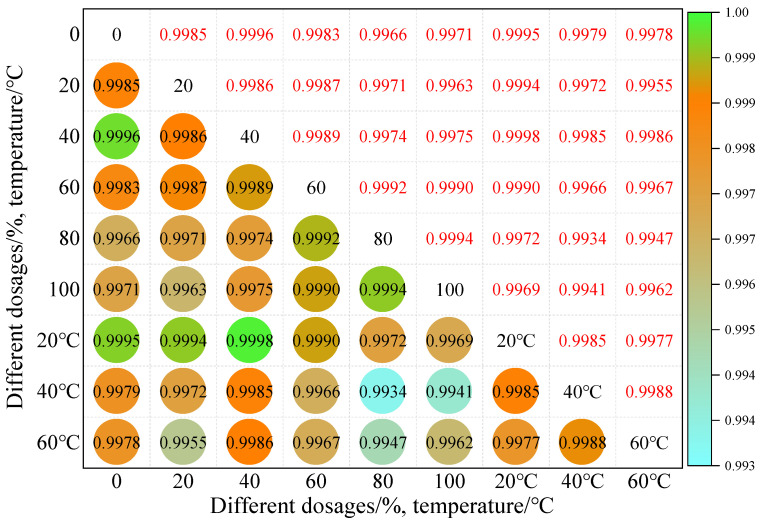
Correlation model of BPN values at different dosages and temperatures.

The effects of different accelerated wear cycles, contents and temperatures on the BPN of iron tailings sand asphalt mixtures have been intuitively reflected in the tests. However, to further reveal the intrinsic correlation between wear action and skid resistance evolution, this study further carried out multi-dimensional correlation analysis. By systematically quantifying the dynamic change characteristics of BPN values under different wear cycles, it not only analyzed the driving mechanism of each factor on the deterioration of micro-texture on the aggregate surface, but also deeply explored the nonlinear correlation and interaction effect with skid resistance attenuation under factor coupling, thus providing key quantitative basis for constructing the skid resistance prediction model based on wear cycles, content and temperature. The results are shown in Figure 9 and Figure 10.

As shown in Figure 9, the inherent correlation and critical deterioration characteristics of the BPN values of iron tailings sand asphalt mixture under different accelerated wear test cycles are revealed. The autocorrelation coefficient of 1.0 on the diagonal line verifies the consistency of the data, while the correlation coefficients in the non-diagonal regions exhibit a phased evolution pattern. When the wear test cycles are within 12,000, the correlation coefficients are mostly positive. However, when the wear test cycles exceed 12,000, the correlation coefficients undergo a significant shift. For example, the correlation coefficient between 0 cycles and 15,000 cycles drops to −0.74, and further decreases to −0.94 between 0 cycles and 18,000 cycles. The microscopic texture on the surface of the aggregate has undergone deep wear, and the BPN value enters a rapid decline zone, marking a critical stage where the risk to traffic safety significantly increases.

It is noteworthy that the correlation coefficient between 15,000 and 18,000 abrasions is as high as 0.90, indicating a strong positive correlation. This reflects that, in the later stage of abrasion, the surface texture of the mixture has entered a stable deterioration phase, with the remaining micro-texture wear rates tending to be consistent. The attenuation of skid resistance gradually decreases, and there are no longer drastic fluctuations as seen in the early stages. In the stable deterioration phase after 15,000 abrasions, the inspection period can be appropriately extended, with a focus on the residual level of texture, in order to achieve the efficient allocation of maintenance resources.

As shown in Figure 10, the intrinsic correlation law of the anti-skid coefficient (BPN) value of iron tailings sand asphalt mixture under different dosages and temperature coupling effects is revealed. The correlation coefficients of all variable combinations are higher than 0.993, showing a strong positive correlation feature, reflecting the universal law of highly synergistic attenuation of anti-skid performance of the mixture. The correlation coefficient between the dosage range of 60% to 80% is generally higher at different temperatures, indicating that the anti-skid performance of this dosage range responds more consistently and stably to temperature changes. The correlation coefficient under the conditions of 20 °C and 40 °C is generally higher than that under 60 °C, and only a relatively low correlation coefficient (0.947) appears under the combination of high dosage (100%) and high temperature (60 °C), revealing that the coupling effect of high dosage and high temperature causes certain differentiation in the evolution law of anti-skid performance.

Based on the above results, it can be clarified that a content of 40%~80% iron tailings sand is more suitable for the medium- and low-temperature (20 °C~40 °C) environment. Under this combination of content and temperature, the anti-slip performance of the mixture shows the best stability and consistency. For high-temperature (60 °C) climate zones, it is recommended to prioritize the use of a dosage of 60% to 80% to avoid the risk of performance differentiation caused by high dosage, while for medium- and low-temperature climate zones, flexible selection can be made within this dosage range to achieve a balance between resource utilization and performance guarantee. However, it should be noted that correlation analysis can only reflect the covariant trend between variables and cannot reveal the inherent logic of the mechanism of action.

#### 3.5.2. Correlation Model of Iron Tailings Sand Content, Abrasion Frequency, and MTD

**Figure 11:**
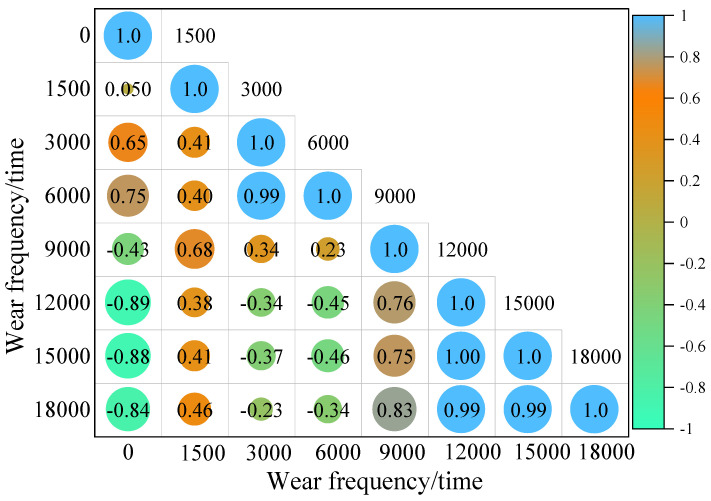
Correlation model of MTD values under different wear cycles.

**Figure 12:**
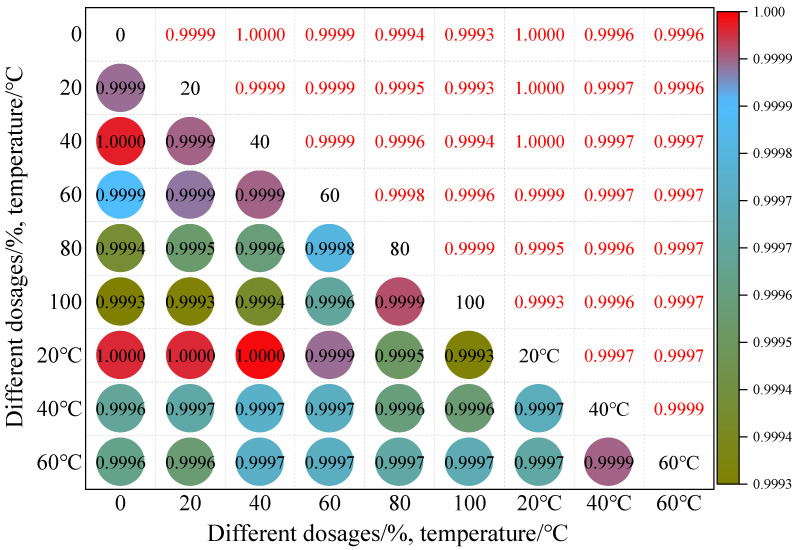
Correlation model of MTD values at different dosages and temperatures.

The effects of different acceleration wear cycles, dosages, and temperatures on the MTD of iron tailings sand asphalt mixtures have been intuitively demonstrated in experiments. However, in order to further reveal the intrinsic relationship between wear and anti-skid performance evolution, this study further conducted multi-dimensional correlation analysis. By systematically quantifying the dynamic changes in MTD values under different wear cycles, not only were the driving mechanisms of various factors affecting the micro-texture degradation of aggregate surfaces analyzed, but also the nonlinear correlations and interactive effects between factor coupling and anti-skid performance degradation were deeply explored. This provides a key quantitative basis for constructing anti-skid performance prediction models based on wear cycles, dosage, and temperature. The results are shown in Figure 11 and Figure 12.

As shown in Figure 11, the inherent correlation and critical deterioration characteristics of the MTD value of iron tailings sand asphalt mixture under different accelerated abrasion cycles are revealed. From the evolution of the correlation coefficient, when the abrasion cycle interval is less than 12,000 times, the correlation coefficient is mostly weakly positive or weakly negative. However, when the abrasion cycle interval exceeds 12,000 times, the correlation coefficient undergoes a significant shift, exhibiting a strong negative correlation. The macro-texture on the aggregate surface has undergone deep wear, and the MTD value enters a rapid decline interval, which is a critical stage where the risk to traffic safety significantly increases. It is worth noting that the correlation coefficient between 15,000 and 18,000 abrasion cycles is as high as 0.99, exhibiting a very strong positive correlation. This reflects that, in the later stage of abrasion, the surface texture of the mixture has entered a stable deterioration stage, and the wear rate of the remaining macro-texture tends to be consistent. The attenuation amplitude of the MTD value gradually narrows, and the previous violent fluctuations no longer occur.

As shown in Figure 12, it systematically reveals the intrinsic correlation law of MTD values of iron tailings sand asphalt mixtures under the coupling effect of different contents and temperatures. The correlation coefficients under all variable combinations are higher than 0.9993, showing an extremely strong positive correlation, which reflects that the deterioration process of the macro-texture of the mixture has high cooperativity and universal regularity. Among them, the correlation coefficients of the 60% content under different temperatures are generally higher than those of other contents, indicating that the texture depth of the mixture with this content has the most consistent response to temperature changes and the optimal macro-texture stability. In contrast, the correlation coefficients under 20 °C and 40 °C are generally higher than those under 60 °C, and a relatively low correlation coefficient only appears in the combination of 100% content and 60 °C, revealing that the coupling effect of high content and high temperature will have a certain impact on the stability of macro-textures.

Based on this, it can be clearly determined that a 60% admixture content is most compatible with medium- and low-temperature (20 °C~40 °C) environments. For high-temperature (60 °C) climatic zones, it is recommended to prioritize the use of a 60% admixture content to avoid the risk of performance differentiation associated with high admixture content. In medium- and low-temperature climatic zones, a flexible choice within the range of 60% to 80% admixture content can be made to achieve a balance between resource utilization and macro-texture stability. However, it should be noted that correlation analysis can only reflect the covariant trend between variables and cannot reveal the inherent logic of the mechanism of action.

## 4. Conclusions

(1) The YIdFert1 model has a good fitting effect on the decay of BPN and MTD (R^2^ ≥ 0.96 for all), and its fitting parameters can be directly used for the long-term prediction of skid resistance under different temperatures; the correlation heatmap reveals the intrinsic correlation and critical characteristics of performance evolution, providing a key basis for constructing the skid resistance prediction model based on wear history. These quantitative achievements can support the skid resistance evaluation and maintenance decision making during the whole life cycle of pavements, and provide systematic theoretical and technical support for the large-scale application of iron tailings sand in asphalt pavements.
(2) Temperature is the core environmental factor affecting the evolution of skid resistance. The iron tailings sand content of 40%~80% is more compatible with the medium- and low-temperature (20 °C~40 °C) environment. Under the combination of this content and temperature, the skid resistance of the mixture shows the best stability and consistency; for high-temperature (60 °C) climate zones, it is recommended to give priority to the content of 60%~80% to avoid the performance differentiation risk caused by high content.
(3) The iron tailings sand content has a significant regulatory effect on the skid resistance attenuation of asphalt mixtures. The asphalt mixture with 60% iron tailings sand content has relatively high initial values and final attenuation values of BPN and MTD, indicating that this content can effectively delay the skid resistance deterioration under long-term wear, thereby delaying the time node of pavement preventive maintenance. Considering the influence of temperature, it is recommended to give priority to the iron tailings sand content of about 60% to configure the asphalt mixture for the anti-skid surface layer in engineering applications.

This study mainly focuses on the evolution characteristics of pavement skid resistance under different replacement contents, temperatures, and abrasion conditions. However, a systematic comparative study on the mechanical properties and pavement performance of asphalt mixtures with various iron tailings sand contents has not been conducted. In addition, a quantitative conversion relationship between indoor accelerated wear cycles and field traffic volume as well as pavement service performance has not been established. We believe that accurate and reliable equivalent conversion must rely on long-term monitoring data from real experimental road sections. Only by comparing and calibrating indoor accelerated wear results with the attenuation of pavement texture and the evolution of skid resistance in the field can a scientific correspondence be established. These are the main limitations of this study.

## Figures and Tables

**Table 1:** Technical indicators of coarse aggregate.

Technical Indicators	Crushing Value/%	Wear Value/%	Polished Value (PSV)	Needle and Flake Particle Content/%	Adhesion to Asphalt (Grade)
Test results	17.1	15.1	63	8.1	5
Technical requirements	≤26	≤28	≥42	≤15	≥4

**Table 2:** Technical indicators of fine aggregate.

Technical Indicators	Crushing Value/%	Wear Value/%	Angularity/S	Needle and Flake Particle Content/%	Apparent Relative Density (g/cm^3^)
Test results	23.5	18.2	42	6.2	2.683
Technical requirements	≤26	≤28	≥30	≤15	≥2.60

**Table 3:** Asphalt technical indicators.

Technical Indicators	Test Results	Technical Requirements
Penetration (25 °C, 100 g, 5 s)/0.1 mm	65	60~80
Softening point (global method)/℃	59	≥55
Extension degree (5 cm/min, 5 °C)/cm	35	≥30
135 °C kinematic viscosity (Pa · S)	2.68	<3

**Table 4:** Technical indicators of iron tailings sand.

Technical Indicators	Technical Requirements	Test Results
Bulk density/g/cm^3^	≥2.50	2.85
Clay content/%	≤3	1.58
Sand equivalent/%	≥60	89
Water absorption rate/%	≤2	0.75

**Table 5:** Gradation design.

Dosage	The Pass Rate of the Following Sieve Holes (%)									
	16	13.2	9.5	4.75	2.36	1.18	0.6	0.3	0.15	0.075
0%	100.0	95.3	68.5	27.1	20.3	18.0	14.0	12.9	11.1	9.8
20%	100.0	95.3	68.5	27.2	21.3	19.3	14.7	13.3	11.3	9.8
40%	100.0	95.3	68.5	27.3	22.5	20.6	15.5	13.9	11.5	9.9
60%	100.0	95.3	68.5	27.5	23.7	21.6	16.1	14.5	11.8	10.1
80%	100.0	95.3	68.5	27.8	25.1	22.6	16.7	15.1	12.1	10.2
100%	100.0	95.3	68.5	28.2	26.5	23.7	17.5	15.7	12.3	10.4

**Table 6:** Marshall test results.

Project	0%	20%	40%	60%	80%	100%
Best oil stone ratio (%)	4.5	4.5	4.6	4.7	4.8	4.9
Relative density of gross volume	2.520	2.521	2.522	2.525	2.539	2.546
Theoretical maximum relative density	2.629	2.630	2.631	2.639	2.655	2.664
Void ratio (%)	4.12	4.14	4.18	4.32	4.43	4.55
Mineral material gap rate (%)	15.29	15.30	15.31	15.35	15.44	15.47
Saturation (%)	72.9	72.8	72.7	71.9	71.4	70.6
Marshall stability (kN)	10.9	10.7	10.6	9.9	9.2	8.3
Flow value (mm)	2.57	2.55	2.56	2.31	2.45	2.75

## Data Availability

The original contributions presented in this study are included in the article. Further inquiries can be directed to the corresponding author.
